# Grading of Recommendations of psychological interventions in the rehabilitation of patients with schizophrenia

**DOI:** 10.1192/j.eurpsy.2024.1541

**Published:** 2024-08-27

**Authors:** R. Vazquez-Noguerol Mendez, S. Garcia Pineiro, P. G. S. Perez, M. Puime Miguez, C. Cinos Galan, M. J. Veiga Candan, M. Suarez Baldomir, M. Soriano Garcia

**Affiliations:** SERGAS, Vigo, Spain

## Abstract

**Introduction:**

As part of the intervention, patients with severe schizophrenia who are cared for in psychiatric rehabilitation units need psychological treatments. However, there is great variability within the psychotherapy alternatives that are proposed for rehabilitation in schizophrenia, and it is necessary to know which are the most efficient interventions in order to prioritize their inclusion in intervention programs.

**Objectives:**

To know the level of evidence of the existing psychotherapy alternatives for the rehabilitation treatment in schizophrenia through the systematic review of recently published studies.

**Methods:**

Consecutive systematic searches in the scientific literature were used in a sensitive and specific way, aimed at identifying the existence of documents in databases and clinical practice guidelines based on evidence of psychological treatment in schizophrenia. Psychosocial and social approaches and family members interventions were excluded, and the search was limited to the last 5 years. The PICO format has been used, and a subsequent critical reading using the AGREE II tool, considering the inclusion criteria of presenting a score >60% in 4 domains.

**Results:**

The following interventions have been found to be therapeutically effective: Level 1B (Early intervention in Psychosis; Patient and Family psychoeducational intervention; Basic ando social skills training; Social cognition and Metakognition training; Cognitive Remediation; Cognitive Behavioral Individual Therapy; Assertive Community treatment; Supported employment). Level 2B (Family Problem Solving Therapy, Dynamic Psychotherapy; Cognitive Behavioral Group Therapy); Level 2C (Horticultural, Art, Music, Animals Therapies).

**Conclusions:**

Several psychotherapy alternatives are proposed for rehabilitation in schizophrenia, with known level of evidenca in order to prioritize their inclusion in intervention programs.

**Disclosure of Interest:**

None Declared

